# Pathological serosa and node-based classification accurately predicts gastric cancer recurrence risk and outcome, and determines potential and limitation of a Japanese-style extensive surgery for Western patients: A prospective with quality control 10-year follow-up study

**DOI:** 10.1054/bjoc.2001.1720

**Published:** 2001-06

**Authors:** D H Roukos, M Lorenz, K Karakostas, P Paraschou, C Batsis, A M Kappas

**Affiliations:** Department of General and Vascular Surgery, Frankfurt Johann Wolfgang Goethe University, Theodor-Stern-Kai 7, 60590 Frankfurt/Main, Germany; Department of Surgery, Ioannina University, Ioannina 451 10, Greece; Department of Mathematics, Ioannina University, Ioannina 451 10, Greece; Department of Biopathology and Laboratory Medicine, Ioannino University Hospital, 45500 Ioannina, Greece

**Keywords:** gastric cancer, extensive surgery, recurrence, survival, prognostic factors

## Abstract

UICC classification accurately predicts overall survival but not recurrence-risk. We report here data of overall and first site-specific recurrence following curative surgery useful for the development of recurrence-oriented preventive target therapies. Patients who underwent resection for gastric cancer were stratified according to curability of surgery [curative (R0) vs non-curative resection], extent of surgery [limited (D1) vs extended (D2) node dissection] and pathological nodal/serosal status. The intent-to-treat principle, log-rank test and Cox regression analysis were used for statistical analysis of time-to-event (recurrence, death) endpoints. Curative resection only produced a chance of cure whereas survival was very poor following non-curative resection (*P *< 0.0001). For D2 R0 subgroup of patients, a pathological serosa and a node state-based classification into three groups, proved to be of clinical implication. Risk of recurrence after a median follow-up of 92 months was low among patients with both serosa and node-negative cancer (first group; 11%), moderate among those with either serosa or node-positive cancer (second group; 53%) and very high among those with both serosa and node-positive cancer (third group; 83%). In multivariate analysis, the relative risks of recurrence and death from gastric cancer among patients in the second and third groups, as compared to those in the first, were 7.07 (95% CI, 2.36–21.17;*P * = 0.0002) and 16.19 (95% CI, 5.76–45.54;*P *< 0.0001) respectively. First site-specific recurrence analysis revealed: low rate of loco-regional recurrence alone (12%), serosa state determinant factor of the site-recurrence (peritoneal for serosa-positive and haematogenous for serosa-negative cancers) and dramatic increase of all types of recurrence by the presence of nodal metastases. Our findings demonstrate that a pathological serosa- and node-based classification is very simple and predicts accurately site-specific recurrence-risks. Furthermore they reveal that risk of recurrence following curative D2 surgery alone is low for serosa- and node-negative cancers, but very high in serosa- and node-positive cancers suggesting the need for new therapeutic strategies in this subgroup of patients. © 2001 Cancer Research Campaign http://www.bjcancer.com


*British Journal of Cancer* (2001) **84**(12), 1602–1609
© 2001 Cancer Research Campaign
doi: 10.1054/ bjoc.2001.1720, available online at http://www.idealibrary.com on IDEAL®

### Pathological serosa and node-based classification accurately predicts gastric cancer recurrence risk and outcome, and determines potential and limitation of a Japanese-style extensive surgery for Western patients: A prospective with quality control 10-year follow-up study

**DH Roukos[1,2], M Lorenz[1], K Karakostas[3], P Paraschou[4], C Batsis[2], and AM Kappas[2]**

[1]Department of General and Vascular Surgery, Frankfurt Johann Wolfgang Goethe University, Theodor-Stern-Kai 7, 60590 Frankfurt/Main, Germany; [2]Department of Surgery, Ioannina University, Ioannina 451 10, Greece; [3]Department of Mathematics, Ioannina University, Ioannina 451 10, Greece; [4]Department of Biopathology and Laboratory Medicine, Ioannino University Hospital, 45500 Ioannina, Greece

**Summary** UICC classification accurately predicts overall survival but not recurrence-risk. We report here data of overall and first site-specific recurrence following curative surgery useful for the development of recurrence-oriented preventive target therapies. Patients who underwent resection for gastric cancer were stratified according to curability of surgery [curative (R0) vs non-curative resection], extent of surgery [limited (D1) vs extended (D2) node dissection] and pathological nodal/serosal status. The intent-to-treat principle, log-rank test and Cox regression analysis were used for statistical analysis of time-to-event (recurrence, death) endpoints. Curative resection only produced a chance of cure whereas survival was very poor following non-curative resection ($P < 0.0001$). For D2 R0 subgroup of patients, a pathological serosa and a node state-based classification into three groups, proved to be of clinical implication. Risk of recurrence after a median follow-up of 92 months was low among patients with both serosa and node-negative cancer (first group; 11%), moderate among those with either serosa or node-positive cancer (second group; 53%) and very high among those with both serosa and node-positive cancer (third group; 83%). In multivariate analysis, the relative risks of recurrence and death from gastric cancer among patients in the second and third groups, as compared to those in the first, were 7.07 (95% CI, 2.36–21.17; $P = 0.0002$) and 16.19 (95% CI, 5.76–45.54; $P < 0.0001$) respectively. First site-specific recurrence analysis revealed: low rate of loco-regional recurrence alone (12%), serosa state determinant factor of the site-recurrence (peritoneal for serosa-positive and haematogenous for serosa-negative cancers) and dramatic increase of all types of recurrence by the presence of nodal metastases. Our findings demonstrate that a pathological serosa- and node-based classification is very simple and predicts accurately site-specific recurrence-risks. Furthermore they reveal that risk of recurrence following curative D2 surgery alone is low for serosa- and node-negative cancers, but very high in serosa- and node-positive cancers suggesting the need for new therapeutic strategies in this subgroup of patients. © 2001 Cancer Research Campaign http://www.bjcancer.com

**Keywords**: gastric cancer; extensive surgery; recurrence; survival; prognostic factors

Despite the well-known universal decline in gastric cancer incidence and mortality, particularly in the USA and Western Europe, stomach cancer remains an important cause of death, worldwide. In Far Eastern countries such as China, Japan, and Korea and also in many developing countries, gastric cancer is the most prevalent malignant neoplasm and the leading cause of cancer death (Roukos, 2000a).

Current reports of treatments for gastric cancer from Japan (Marujama et al, 1987; Fujii et al, 1999), and Korea (Kim, 1999), when compared to historical data, suggest a marked improvement in the overall survival rates. This improvement is attributable to an earlier detection and, according to Eastern investigators, to a more radical surgical approach (Marujama et al, 1987; Fujii et al, 1999, Kim et al, 1999). In Japan, due to a mass screening programme, the rate of early gastric cancer that is associated with a good prognosis, has increased to 50% and curative resection is possible in up to 93% of cases (Fujii et al, 1999), whereas these same figures in the West are only about 15% and 50 to 70% respectively (Roukos, 2000a). Some Western authors support the notion that the better survival results in the East are exclusively attributable to the early detection of the disease, and not to the more aggressive surgery that is employed in the East (Bonenkamp et al, 1999).

A surgical resection with curative intent, R0 resection according to the International Union Against Cancer (UICC) (Beahrs et al, 1992), is the treatment of choice for gastric cancer; in that the prognosis for all other patients with apparently residual tumour following surgery is extremely poor. However, even after an R0 resection, the proportion of treatment failures for advanced gastric cancer is substantially high. For the improvement of both local control and survival, a more extensive surgery is proposed. However, it is still unclear whether curative resection should include, besides gastrectomy, an extended lymph node dissection as occurs in the East. Randomized controlled trials in the West

*Received 13 September 2000*
*Revised 24 January 2001*
*Accepted 25 January 2001*

*Correspondence to:* DH Roukos have failed to demonstrate a survival benefit in favour of D2 dissection (Bonenkamp et al, 1999; Cuschieri et al, 1999), but there is still uncertainty because of strong criticisms of these trials (Brennan, 1999; Roukos, 2000b).

The assessment of the gastric cancer recurrence risk and outcome is an important and urgent issue, however, cancer and especially gastric cancer is complicated by the fact that recurrence occurs in a variety of forms and in different organs following R0 resection; loco-regional, peritoneal, lymph nodal, haematogenous and combinations of these, constitute the major sites of recurrence. Therefore, the establishment of an extensive Japanese-style surgery for the control of the disease in Western patients is of practical relevance and of even greater social significance because no adjuvant treatment, that would contribute to survival improvement, has yet been established to be effective (Hermans et al, 1993; Roukos, 2000a). The target of this prospective study, was to assess the effectiveness of a standardized extensive surgery, by evaluating risk factors for recurrence and the overall survival of patients who had previously undergone this surgical approach at a median follow-up of 92 months.

### PATIENTS AND METHODS

All consecutive patients with a histologically confirmed gastric carcinoma who had undergone resection between January 1986 and December 1992 were included in this prospective study. We excluded patients who had a palliative surgical procedure without gastrectomy because resection is necessary for accurate histological diagnosis and staging. Patients were also excluded if they had a previous or coexisting cancer.

After surgery, the patients underwent a clinical examination, laboratory tests, X-rays, endoscopic or radiologic examination and abdominal ultrasound every 3 months or computer tomography every 6 months in the first 2 years. Thereafter, all these examinations were performed every 6 months.

### Surgery

The guidelines of the Japanese Research Society for Gastric Carcinoma (JRSGC), for the standardization of surgical treatment and pathological evaluation (Nishi et al, 1995), as well as the recommendations by the American Joint Committee on Cancer (AJCC) and the UICC in the fourth edition of their manual for the staging of cancer (Beahrs et al, 1992), formed the basis of our protocol. Total gastrectomy with an extended lymph-node dissection was the treatment of choice. A subtotal gastrectomy (SG) was only performed in patients with an early stage (T1) intestinal-type growth pattern, according to the Lauren classification, or in older patients not in good physical condition. Extended (D2) lymph node dissection was performed using a systematic and standardized technique according to the guidelines of the JRSGC. D2 node dissection in our study entailed the removal of perigastric compartment I nodes (stations 1 to 6, attached to the stomach: D1 dissection) and the extraperigastric compartment II nodes (stations 7 to 12) using a technique previously described (Roukos et al, 1998). The total gastrectomy specimen with greater and lesser omenta and containing nodal stations 1 through 6, including the nodes along the left gastric artery (station 7) was removed en-block and sent to the pathologist. However, the fatty connective tissue containing nodes from each of the 3 major compartment II nodal areas, i.e., hepatoduodenal ligament (station 12), superior border of the pancreas (stations 8, 9 and 11), and spleen hilus (station 10), was separately dissected, labeled and sent to the pathologist. Resection of the spleen was optional.

### Pathology and quality control

All histopathological data were prospectively documented in a standardized protocol. The pathological lymph node grouping (pN) was done according to the rules of the JRSGC slightly modified in our protocol pN1 stage: detection of metastasis by pathologist in compartment I lymph nodes (stations 1 through 6), but no metastases in compartment II; pN2: metastases in compartment II nodes (stations 7 through 12) but no metastases in compartment III. For tumour invasion (pT), distant metastases (pM) and curability of resection, the recommendations of the AJCC/UICC were applied.

Obviously, the risk of recurrence should only be studied among patients who had a complete resection in the apparent absence of remaining disease. Because the curability of resection includes, besides the resection of the primary tumour, the dissection of metastatic nodes, we evaluated the risk of relapse among patients who had an extended (D2) node dissection, which ensures an accurate nodal staging. An objective estimation of the risk of relapse among patients who had undergone a limited (D1) dissection appears to be unreliable because a substantial proportion of these patients have remaining disease after a D1 dissection. This because it has been shown that compartment II nodes that are left behind after a D1 resection, are tumour positive in 30% of patients who had a D2 resection with curative intent (Bunt et al, 1995; Roukos, 2000a, 2000b).

We used the pathological and surgical findings documented in the protocol to stratify patients according to the curability of resection (curative (R0) or noncurative (R1, R2)) and extent of lymph-node dissection (D1 or D2) based on standardized criteria. Patients were classified as having a curative resection if, at laparotomy, there was no macroscopic evidence of hepatic or peritoneal spread of the tumour or metastatic deposits beyond the compartment II nodes, that the resection resulted in complete macroscopic tumour removal and in the final pathological examination there was no microscopic evidence of tumour cells in all resection lines. The patients who met these macroscopic and microscopic criteria constituted the group treated curatively (R0 resection), all the others who did not meet these criteria constituted the group treated with a palliative intent (R1, R2 resection).

To control the surgical report as to whether a D2 node dissection was completely performed, we used the pathology report from the lymph-node examination. For the quality control, the number of retrieved lymph-nodes per station by the pathologist and the intrinsic biological variation of nodes per station (Marujama et al, 1987) were considered. Details for this quality control are described in an our recent report (Roukos et al, 2000).

It should be noted that none of the patients treated curatively underwent adjuvant chemotherapy or radiotherapy and thus the surgery alone is responsible for the reported results.

### Statistical analysis

All diagnostic, surgical and histopathological data and records of events (death, recurrence or censored patients) were prospectively documented on a standardized protocol. The primary endpoints were recurrence-free survival and overall survival, as measured

*© 2001 Cancer Research Campaign*

from the date of surgery to the time of the last follow-up visit or death, irrespective of cause. The treatment effect was primarily evaluated according to the intention-to-treat principle and all the patients were included in the overall survival analysis, irrespective of whether they had an R0 or R1, R2 resection. Because the survival of patients who had noncurative surgery is extremely poor and cannot be affected by any type of surgical or adjuvant treatment, all subsequent analyses were focused on the patients who had undergone an R0 resection. Only the patients who had not died in the hospital after a D2 curative resection were at risk of recurrence, we therefore only estimated the risk of recurrence amongst these patients.

We constructed Kaplan–Meier life-table curves in order to estimate the probability of treatment failure for the endpoints of disease-free survival and overall survival (Kaplan and Meier, 1958), and used the log-rank test for comparison. Data on patients who were alive and had no evidence of disease at the end of our study or at the last follow-up visit were censored. We used a multivariate Cox proportional-hazards analysis to estimate the prognostic effect of various variables with respect to relapse-free survival and overall survival (Cox, 1972). A $P$ value of less than 0.05 was considered to indicate statistical difference. For statistical analyses, we used SPSS software for Windows (version 9.0.1).

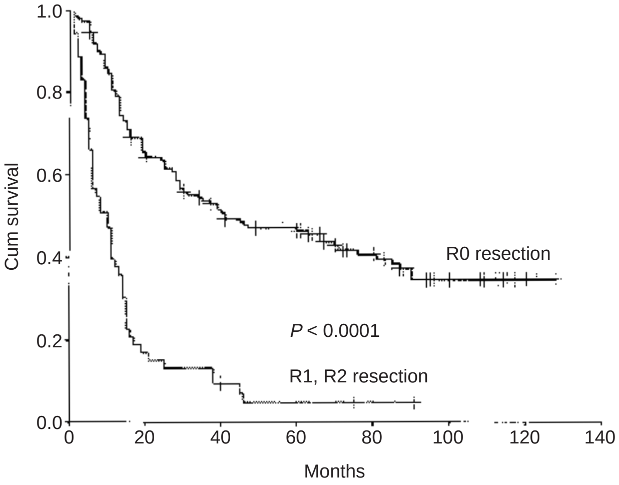

**Figure 1** Kaplan–Meier survival curves for patients with gastric cancer stratified according to the curability of resection. The survival of patients who had a curative resection (R0) was significantly better than that of patients who had a non-curative resection (R1, R2) ($P < 0.0001$)

### RESULTS

Of the 210 patients with gastric carcinoma who underwent gastrectomy, 59 (28%) had a non-curative resection and 151 (72%) underwent a curative resection. Of these 151 R0 patients, our criteria for a D2 node dissection were fulfilled by the 124 patients (79%).

### Long-term survival for all resected patients

All resected patients were included in the primary analysis of overall survival according to the intention-to-treat principle, irrespective of curability (R0, R1/R2 resection). Of 210 resected patients, 202 (96%) could be followed. During a median follow-up of 54 months, 139 patients died and 63 were alive without evidence of recurrence (31.2%). The curability of resection (curative vs. noncurative, $P < 0.001$, Figure 1), nodal status (pN-stage, $P < 0.0001$), tumour invasion (pT-stage, $P < 0.0001$) and extent of surgery (extended vs. limited lymph-node dissection, $P < 0.0001$) were all significant prognostic factors according to the log-rank test in univariate analysis. In a step-down multivariate analysis, the nodal status ($P < 0.0001$) and the curability of resection ($P = 0.0001$) were found to be the strongest significant and independent predictors of outcome.

Further analysis of the results according to the curability of resection shows that the median survival time following noncurative resection was very poor (8 months). The relative risk of death among palliatively resected patients, as compared with those who had a curative resection, was 2.32 (95% confidence interval, 1.53 to 3.52). Since the prognosis of patients with residual disease after resection was extremely poor, and that this dismal survival unfortunately cannot be improved by any type of surgery, we estimated the effect of extensive surgery on loco-regional or any type of recurrence and survival on patients who had a D2 resection with curative potential.

### Survival of patients who had a D2 curative lymph-node dissection

Table 1 shows the clinical and tumour characteristics. Most of patients (91%) had a total gastrectomy and an advanced pathological T3-cancer (53%). Of the 124 patients who fulfilled our criteria for a D2 resection with curative intent, there were only 2 postoperative in-hospital deaths (1.6%). One patient was lost to follow-up (0.8%). Of the 121 patients who left the hospital and thus were at risk of relapse, during a median follow-up of 92 months for survivors, gastric cancer recurred in 59 patients (49%). The median time from surgery to the evidence of recurrence or death from gastric cancer for these 59 patients was 10 months (73% recurred within the first two years and only 5% after the fifth year; mean survival time 17.24 months (range 1 to 70). These 59 patients died shortly after the first evidence of recurrence (median time, 5 months (range 0 to 33)).

Table 2 shows the sites of first recurrence and status of patients at their last follow-up according to the serosa and lymph node state. The frequency of any type of recurrence increased with serosa invasion and the presence of lymph-node metastases. There was no difference between the intestinal-type and diffuse-type cancer according to the Lauren-classification in any type of recurrence. Evidence of the first site of relapse was obtainable in 34 patients (58%), whereas it was impossible in the remaining 25 patients because of the rapid progression of the disease from surgery to death with multiple metastases in different organs. Among these 34 patients, loco-regional recurrence as the only cause of a first relapse, was rarely assessed (12%), whereas peritoneal or haematogenous recurrences were more frequently evident (88%). Peritoneal dissemination as the only site of recurrence was the most frequent treatment failure (29%).

There was a strong correlation between serosa state and the type of recurrence. Peritoneal recurrence occurred exclusively among patients with serosa invasion, whereas none of the serosa-negative cancers had a peritoneal relapse. This figure persisted even though we calculated all patients who had peritoneal failure alone or in combination with other organ recurrences. Of the 20 patients with peritoneal recurrence alone or in combination with other organs, 19 had a serosa invasion (95%) and only one (5%) had a serosa-negative cancer (pT2 tumour). Interestingly, in 88% of the

 © 2001 Cancer Research Campaign patients, the evidence of first recurrence was exclusively confined to the abdominal cavity. However, 29% of these patients had multiple recurrences in different organs (peritoneal surface, liver, local) (Table 2).

Both the lymph-node status (pN category) and tumour invasion (pT category) were found to be significant factors in the prediction of both relapse-free survival and overall survival, whereas the location of the tumour and the Lauren classification had no prognostic relevance in univariate analysis (Table 3). In a Cox multivariate regression analysis, nodal status and tumour invasion were each found to be significant and independent predictors of both recurrence and gastric cancer-related death ($P$ = 0.002 and $P$ = 0.005, respectively), and of death of any cause ($P$ = 0.002 and $P$ = 0.02 respectively) (Table 4).

### Pathological serosa and node state-based risk-prediction analysis

Since microscopic nodal and wall invasion (pN, pT categories) were found to be independent predictors of survival and that there was a strong correlation between the serosa status and site of first recurrence, we performed a recurrence and death risk-prediction analysis with a combination of both prognostic variables to assess whether this combination could more accurately predict both the risk of relapse and death from any cause. From the stratification of the patients according to both serosa and nodal state derived 4 groups: serosa-, and node-negative cancers ($pT_{1,2}N_0$, first group), serosa-positive but node-negative cancer ($pT_3N_0$, group B1), serosa-negative but node-positive cancer ($pT_{1,2}N_{1,2}$, group B2) and serosa- and node-positive cancers ($pT_3N_{1,2}$, third group). It was found that there was no significant difference between the patients in groups B1 and B2 with respect to either relapse-free survival ($P$ = 0.72) or overall survival ($P$ = 0.38) and thus these 2 subgroups were included in the same group (second group; $pT_3N_0$, $pT_{1,2}N_{1,2}$).

Of 47 patients in the third group, 39 recurred and died (83%), whereas of 32 in the second group, 16 recurred and died (50%). Among 42 patients in the first group only 4 recurred and died (10%) (Table 2). As shown in Figure 2, after 10 years of follow-up, patients in the first group were at low risk (11%), those of second group at high risk (53%) and those of the third group at very high risk (83%) of recurrence and death from gastric cancer. The differences between the 3 groups in both risk of recurrence and death from gastric cancer or death from any cause were, by log-rank test, highly significant ($P$ < 0.0001).

We performed a Cox multiple-regression analysis, entering these 3 groups into the model (Table 5). This classification accurately predicted both the cumulative risks of recurrence and death from gastric cancer and of death from any cause. The relative risks of recurrence and death from gastric cancer among patients in the second and third groups, as compared to that in first group, were 7.07 (95% confidence interval (CI, 2.36–21.17; $P$ = 0.0005) and 16.19 (95% CI, 5.76–45.54; $P$ < 0.0001) respectively).

**Table 1** Characteristics of 124 patients who underwent a D2 resection with curative potential (R0)

| Variable | No. of patients | percent |
|---|---|---|
| All patients | 124 | |
| Median age (range) | 65 (29–86) | |
| Sex (M/F) | 77/47 | |
| Location of tumour | | |
| Cardia, upper third | 34 | 27.5 |
| Middle third | 41 | 33 |
| Distal third | 41 | 33 |
| More than two thirds | 8 | 6.5 |
| Depth of invasion (UICC/AJCC)[a] | | |
| Serosa-negative cancers | 58 | 46.8 |
| pT1 | 25 | 20.2 |
| pT2 | 33 | 26.6 |
| Serosa-positive cancer (pT3) | 66 | 53.2 |
| Lymph node status (JRSGC)[b] | | |
| Node-negative cancers (pN0) | 60 | 48.4 |
| Node-positive cancer | 64 | 51.6 |
| pN1 | 33 | 26.6 |
| pN2 | 31 | 25 |
| Lauren classification | | |
| Intestinal | 48 | 38.7 |
| Diffuse or mixed type | 76 | 61.3 |
| Type of gastrectomy | | |
| Total | 113 | 91 |
| Subtotal | 11 | 9 |
| Resection of spleen | 67 | 54 |
| Resection of tail of pancreas | 7 | 5.6 |

[a]The tumour-node-metastasis classification of the Union International contre le Cancer (UICC) and the American Joint Committee on Cancer, 4th edition was used.[6]
[b]The nodal stage of the Japanese Research Society for Gastric Cancer, 1st English edn. was used.[12] The abbreviation pT, pN denotes pathologically confirmed tumour-nodes.

### DISCUSSION

Our results confirm that curative resection (R0) is a very strong and independent predictor of outcome for gastric cancer. The survival for all other patients with remaining disese after resection is extremely poor and cannot be improved by any type of treatment, thus our study focused on patients who had an R0 resection. Although surgery with curative potential is without doubt the treatment of choice, controversy still remains as to the optimal extent of this resection. The hypothesis for the improvement of both local control and survival and the favourable findings with respect to an extended lymph node dissection in reports from East (Marujama et al, 1987; Fujii et al, 1999; Kim, 1999), and West (Siewert et al, 1998), have not been confirmed by 2 recent European randomized trials (Bonenkamp et al, 1999; Cuschieri et al 1999). Controlled trials are the best method for making treatment decisions (Sackett et al, 1996), but criticism regarding their appropriateness of design and conduct (Brennan, 1999; Roukos, 2000b). Have lead now to an uncertainty over the optimal extent of surgery. We conducted this study in order to assess the potential of a Japanese-type radical surgery in Western patients. Because experience and pancreas preservation are predominant factors for both the safety and completeness of an extended lymph node dissection (Siewert et al, 1998; Brennan, 1999; Roukos, 2000b), we started this study after gaining 7 years experience with D2 dissection (Roukos et al, 1990). This strategy and the low rate of pancreatectomies explains the low rate of in-hospital mortality (1.6%) in our study, which is similar to that of other reports (Marujama et al, 1987; Siewert et al, 1998; Fujii et al, 1999; Kim, 1999) suggesting the safety of the D2 procedure when the criteria of a surgeon's experience and pancreas-preserving technique are met. The low rate of in-hospital mortality with a high rate of prospective

*© 2001 Cancer Research Campaign*

**Table 2** Types of recurrences and status of patients at last follow-up according to serosa and lymph nodes state of patients who had undergone a curative D2 resection

| Site of relapse | No. of patients | Numbers of patients (percent) | | | |
|---|---|---|---|---|---|
| | | Serosa-negative cancer (pT1, T2) | | Serosa-positive cancer (pT3) | |
| | | Node-negative (pN0) | Node-positive (pN1, N2) | Node-negative (pN0) | Node-positive (pN1, N2) |
| Patients at risk of relapse | 121 | 42 (35) | 15 (12) | 17 (14) | 47 (39) |
| Recurrences | 59 (49) | 4 (10) | 8 (53) | 8 (47) | 39 (83) |
| First site undefined | 25 (42) | 2 | 6 | 1 | 16 |
| First site clearly defined | 34 (58) | 2 | 2 | 7 | 23 |
| Loco-regional alone | 4 (12) | 0 | 1 | 1 | 2 |
| Distant metastases | 30 (88) | 2 | 1 | 6 | 21 |
| Peritoneal alone | 10 (29) | 0 | 0 | 1 (14) | 9 (39) |
| With local | 3 | 0 | 0 | 2 | 1 |
| With liver | 7 | 1 | 0 | 1 | 5 |
| Liver alone | 6 (11) | 0 | 0 | 2 | 4 |
| Extra-abdominal alone or with intra-abdominal failure | 4 (20) | 1 | 1 | 0 | 2 |
| Status of patients at last follow-up | | | | | |
| All patients | 124 | 42 | 16 | 18 | 48 |
| Alive | | | | | |
| Without recurrence | 51 (41) | 33 (79) | 4 (25) | 8 (44) | 6 (13) |
| With recurrence | 0 | | | | |
| Dead | 72 | 9 (21) | 12 (75) | 10 (56) | 41 (87) |
| Recurrence | 59 (49) | 4 (10) | 8 (53) | 8 (47) | 39 (83) |
| In-hospital postoperatively | 2 (1.6) | 0 | 1 | 1 | 0 |
| Cause other than gastric cancer | 11 (10) | 5 | 3 | 1 | 2 |
| Lost to follow-up | 1 (0.8) | 0 | 0 | 0 | 1 |

[a]First site of recurrence could not be diagnosed due to rapid progression with multiple recurrences in different organs.

**Table 3** Univariate analysis of predictive factors for relapse and death or death from any cause in patients with gastric cancer who had a curative D2 resection

| Variable | Relapse-free survival[a] *n* = 121 | | | | Overall survival[b] *n* = 123 | | | |
|---|---|---|---|---|---|---|---|---|
| | No. of Patients Events/Total | 5-Yr % | 10-Yr % | *P* Value[c] | No. of Patients Events/Total | 5-Yr % | 10-Yr % | *P* Value[c] |
| Location of tumor | | | | 0.10 | | | | 0.25 |
| Upper third stomach | 17/34 | 48 | 48 | | 21/34 | 41 | 35 | |
| Middle third | 18/40 | 57 | 53 | | 23/40 | 50 | 40 | |
| Distal third | 17/39 | 57 | 53 | | 21/41 | 51 | 44 | |
| More than two thirds | 7/8 | – | – | | 7/8 | – | – | |
| Lauren classification | | | | 0.42 | | | | 0.79 |
| Intestinal-type cancer | 20/46 | 55 | 51 | | 28/47 | 44 | 35 | |
| Diffuse or mixed type | 39/75 | 50 | 47 | | 44/76 | 52 | 39 | |
| Tumour invasion[d] | | | | <0.0001 | | | | <0.0001 |
| pT1 | 2/24 | 91 | 91 | | 4/25 | 88 | 82 | |
| pT2 | 10/33 | 64 | 64 | | 17/33 | 53 | 44 | |
| pT3 | 47/64 | 31 | 26 | | 51/65 | 32 | 19 | |
| Lymph-node status[e] | | | | <0.0001 | | | | <0.0001 |
| pN0 | 12/59 | 80 | 78 | | 19/60 | 74 | 64 | |
| pN1 | 22/33 | 31 | 31 | | 25/33 | 33 | 23 | |
| pN2 | 25/29 | 19 | 10 | | 28/30 | 17 | 5 | |

Of 124 patients, one was lost to follow-up. [a]Patients who left the hospital were at risk of recurrence. This analysis included all 121 patients who did not die in hospital; 2 in-hospital deaths were excluded. [b]This analysis included all 123 patients who were at risk of death due to any cause including the 2 in-hospital deaths. [c]The log-rank test was used. [d]Pathological tumour stage according to UICC/AJCC system.[6] [e]Pathological nodal stage according to JRSGC classification.[12]

*© 2001 Cancer Research Campaign*

**Table 4** Cox multivariate proportional-hazards analysis for patients who had a D2 resection with curative intent[a]

| Variable | Relapse and death[b] *n* = 121 | | Death from any cause[c] *n* = 123 | |
|---|---|---|---|---|
| | ***P* value** | **Relative risk (95% CI)** | ***P* value** | **Relative risk (95% CI)** |
| Lymph-node status[d] | 0.002 | | 0.002 | |
| pN1 vs pN0 | 0.009 | 2.70 (1.28–5.73) | 0.01 | 2.20 (1.16–4.19) |
| pN2 vs pN0 | 0.0005 | 3.77 (1.78–7.99) | 0.0004 | 3.23 (1.68–6.19) |
| Tumour invasion[e] | 0.005 | | 0.02 | |
| pT2 vs pT1 | 0.19 | 2.81 (0.59–13.40) | 0.08 | 2.73 (0.89–8.44) |
| pT3 vs pT1 | 0.01 | 6.81 (1.51–30.84) | 0.01 | 4.18 (1.38–12.67) |

[a]Of 124 patients, one was lost to follow-up. [b]This analysis included all 121 patients who were at risk of relapse; 2 in-hospital deaths were excluded. All 59 patients who relapsed died shortly after the evidence of recurrence (median time, 5 months). [c]This analysis included all 123 patients who were at risk of death of any cause including the 2 in-hospital deaths. [d]Pathological nodal stage (pN) according to JRSGC classification.[12] [e]Pathological tumour stage (pT) according to UICC/AJCC system.[6] The abbreviation CI denotes the confidence interval.

**Table 5** Cox-multivariate proportional-hazards analysis of patients who had a curative D2 resection stratified according to both the serosa and node state[a]

| Variable | Recurrence and death[b] *n* = 121 | | Death from any cause[c] *n* = 123 | |
|---|---|---|---|---|
| | ***P* value** | **Relative risk (95% CI)** | ***P* value** | **Relative risk (95% CI)** |
| Pathological serosa and node state[d] | <0.0001 | | <0.0001 | |
| Either serosa or node-positive ($pT_3$ $N_0$ or $pT_{1,2}$ $N_{1,2}$) vs both negative ($pT_{1,2}$ $N_0$) | 0.0005 | 7.07 (2.36–21.17) | 0.0002 | 4.32 (1.99–15.27) |
| Both serosa and node-positive ($pT_3$ $N_{1,2}$) vs both negative ($pT_{1,2}$ $N_0$) | <0.0001 | 16.19 (5.76–45.54) | <0.0001 | 7.35 (3.55–15.21) |

[a]Of 124 patients, one was lost to follow-up. [b]Patients were stratified according to the pathological evidence of serosa invasion (pT3) or not (pT1, pT2) and the presence of lymph-node metastases (pN1, pN2) or not (pN0). Pathological tumour staging was done according to UICC/AJCC system, 4th edition,[6] and nodal staging (pN) according to JRSGC.[12] [c]This analysis included all 121 patients who were at risk of relapse; the 2 in-hospital deaths were excluded. All of the 59 patients who relapsed died shortly after the evidence of recurrence (median time, 5 months). [d]This analysis included all 123 patients who were at risk of death through any cause including the 2 in-hospital deaths.

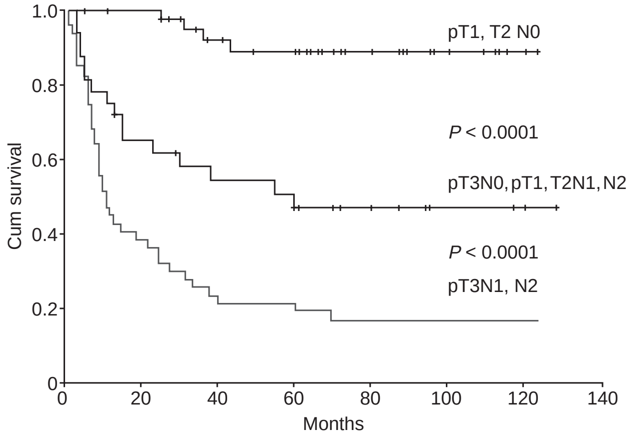

**Figure 2** Kaplan–Meier relapse-free survival curves for patients with gastric cancer, stratified according to both the pathological serosa- and node-state based classification. The differences in the cumulative risks of recurrence and death from gastric cancer between the 3 groups of patients with both serosa- and node-negative cancer (first group; $pT_{1,2}$ $N_0$) or either serosa- or node-positive cancer (second group; $pT_3N_0$, $pT_{1,2}$, $N_{1,2}$) or both positive cancer (third group; $pT_3$ $N_{1,2}$) were highly significant (*P* < 0.0001)

documentation of all events, allows a follow-up in nearly all of the patients enrolled in the study.

UICC/AICC classification predicts accurately overall survival but is not able to predict overall recurrence and site-specific recurrence-risks. A recurrence-risk prediction analysis on patients who had an R0 resection is important in determining both the potential and limitations of surgery and therefore in making decisions about the need for adjuvant treatment. Our study confirms the predictive value of tumour depth (pT-category) and nodal status (pN-category); however, both a pathological serosa and node state-based classification of patients into 3 major subgroups proved to be of clinical significance for 3 reasons.

Firstly, this classification was highly accurate in predicting recurrence-risk and death among patients who had either both serosa and node negative cancer (first group, low risk; 11%) or both positive (third group, very high risk; 83%). The difference in the risk of treatment failure between these two groups was highly significant in multivariate analysis (relative risk 16.19, 95% CI 5.76–45.54; p < 0.0001). However, in the second group of patients with either serosa- or node-positive cancer, this predictive value was not high (53%).

Secondly, a serosa and node state-based classification proved useful in predicting the site of first recurrence-risk. Obtaining evidence for the first site of recurrence in gastric cancer is challenging because it occurs rapidly after an R0 resection in a variety of forms and in different organs (Maehara et al, 2000). Indeed, we were only able to provide evidence of the first site of failure in only 58% of cases. An intensive monitoring for increased early detection of first site recurrence is of no practical value since the dismal prognosis of recurrent gastric cancer can not be altered by any type of treatment. Despite these difficulties, the data contained within our study and that of other reports (Averbach and Jacquet, 1996; Nakajima et al, 1999; Maehara et al, 2000), clearly indicate a strong correlation between the serosa state and peritoneal recurrences. This is the most common type of treatment failure among serosa-positive patients, but it rarely occurs among serosa-negative patients for whom a haematogenous recurrence is the most frequent.

Thirdly, the potential of surgery proved to be closely related to the serosa and node state-based classification. Surgery alone resulted in excellent survival for most patients with both serosa-and node-negative cancer. These patients had a very good prognosis (mean overall survival time 103 months; ± 6; 95% CI 91 to 114 months), similar to that of Eastern reports (Marujama et al, 1987; Fujii et al, 1999; Kim, 1999), suggesting the reproducibility of Japanese results in the West for this subgroup of patients. A further improvement of outcome with adjuvant treatment appears, at present, unrealistic. The ineffectiveness of adjuvant chemotherapy (mitomycin, fluorouracil, uracil, tegafur) that was proven in a recent controlled trial for serosa-negative cancer and a possible adverse effect of drugs on the host immune surveillance system (Nakajima et al, 1999) do no support the use of adjuvant chemotherapy in these patients. However, surgery was proven to be less effective in the 2 other groups, accounting for 65% of the total of our R0 patients. At 10 years after surgery, 32% and 12% only of patients were alive in the second and third groups respectively. These survival rates are substantially lower compared to the Japanese reports. The likely explanation for this treatment difference is still unclear, although it has been suggested to be attributable to stage migration according to Western investigators (Bonenkamp et al, 1999) or to a more extensive surgery resulting in a better control and survival according to Japanese investigators (Fujii et al, 1999). Indeed, we assessed that overall, there was a low rate of loco-regional recurrence (12%), which was distinctly lower compared to reports from USA with limited surgery (40%) (Wanebo et al, 1993), but overall survival was poor. These findings suggest the potential of extensive surgery in reducing loco-regional failure, but clearly indicate its limitation in affecting distant recurrences and clinical outcome. Theoretically, the most likely explanation for this high treatment failure rate among patients with advanced gastric cancer who had a surgically complete tumour resection, is the presence of undetected disseminated tumour cells or distant micrometastases at the time of surgery. This view is strongly supported by the low rate of local failure and an early distant recurrence after an R0 resection in our study. The systemic component of the disease has been demonstrated even in its early stages, at best for breast cancer (Braun et al, 2000), and shown to be stage-dependent. However, the prognostic significance of these findings is still to proven (Zippelins et al, 2000).

Ideally, the establishment of the prognostic value of disseminated tumour cells and that of biologic new tumour prognostic factors may lead to a more effectively tailored therapy towards individual patients. A sophisticated staging would also help us to understand the critical question why some patients develop recurrences and others do not, although they had similar clinical and conventional tumour characteristics and had underwent the same appropriate treatment. The systemic component of the disease and the ineffectiveness of postoperative, late chemotherapy administration (Hermans et al, 1993; Averbach and Jacquet, 1996; Roukos, 2000a), justifies the research interest focused in testing the efficacy of neoadjuvant (preoperative) or intraoperative intraperitoneal chemotherapy for the prevention of haematogenous or peritoneal surface recurrences in controlled ongoing trials.

At present, however, a pathological serosa and node-based classification into 3 groups is simple, feasible, and realistic in all institutions. The high value in predicting first site and overall recurrence and outcome, the highly significant differences between the 3 groups, and the determination of potential and limitations of extensive surgery in a strong relation to this classification, establish it as the key point in daily clinical practice. However, the validity and the accuracy prediction rate of this classification is strongly dependent, from the careful, prospective documentation of all clinicopathologic data and on a quality control in stratification of patients according to curability of resection (R0 or R1, R2) and extent of lymph node dissection (D1 or D2). The appropriate surgery for patients with serosa and node-negative cancer, leads to a very good survival rate and there seems to be no further need for adjuvant treatment. However, in the 2 other patient groups, survival rates are substantially reduced in a serosa and node stage-dependent manner. Occult disseminated tumour cells at the point of surgery are likely to be responsible for high recurrence rates and poor survival, thus indicating the limitations of surgery in advanced gastric cancer. Therefore, there is an urgent need for an adjuvant treatment, but despite great efforts over the last 3 decades, no effective chemotherapy regimen has been established. An exaggeration in the interpretation in the outcome for patients with advanced gastric cancer, or, an overoptimism as expressed recently with the phrase ‘End to cancer in sight...’ is criticized as being unrealistic; to expect a massive reduction in the mortality rate on the basis of results of new drugs is also premature, as has been pointed out in a Lancet Editorial (Lancet, 2000). At present, early detection and appropriate treatment may contribute to a substantial reduction in case-fatality in gastric cancer.

### REFERENCES

Averbach AM and Jacquet P (1996) Strategies to decrease the incidence of intra-abdominal recurrence in resectable gastric cancer. *Br J Surg* **83**: 726–733

Bearhs OH, Henson DE, Hutter RVP and Kennedy BJ (eds) (1992) *Manual for staging*. American Joint Committee on Cancer. 4th edition. Philadellphia: J. B. Lippincott

Bonenkamp JJ, Hermans J, Sasako M and van de Velde CJH (1999) Extended lymph-node dissection for gastric cancer. *N Engl J Med* **340**: 908–914

Braun S, Pantel K, Muller P, Janni W, Hepp F, Kentenich CR, Gastroph S, Wischnik A, Dimpfl T, Kindermann G, Riethmuller G and Schlimok G (2000) Cytoceratin-positive cells in the bone marrow and survival of patients with stage I, II, or III breast cancer. *N Engl J Med* **342**: 525–533

Brennan MF (1999) Lymph-node dissection for gastric cancer. *N Engl J Med* **340**: 956–958 (editorial).

Bunt AMG, Hermans J, Smit VTHBM, van de Velde CJH, Fleuren GJ and Bruijn JA (1995) Surgical/pathologic-stage migration confounds comparison of gastric cancer survival rates between Japan and Western countries. *J Clin Oncol* **13**: 19–25

Cox DR (1972) Regression models and life tables. *J R Stat Soc [B]* **34**: 187–220

Cuschieri A, Weeden S, Fielding J, Bancewicz J, Craven J, Joypaul V, Sydes M and Fayers P (1999) Patient survival after D1 and D2 resection for gastric cancer: long-term results of the MRC randomised surgical trial. Surgical co-operation group. *Br J Cancer* **79**: 1522–1530

*© 2001 Cancer Research Campaign*

Hermans J, Bonenkamp JJ, Boon MC, Bunt AM, Ohyama S, Sasako M and Van de Velde CJ (1993) Adjuvant therapy after curative resection for gastric cancer: meta-analysis of randomized trials. *J Clin Oncol* **11**: 1441–1447

Fujii M, Sasaki J and Nakajima T (1999) State of the art in the treatment of gastric cancer: from the 71st Japanese Gastric Cancer Congress. *Gastric Cancer* **2**: 151–157

Kaplan EL and Meier P (1958) Nonparametric estimation from incomplete observations. *J Am Stat Assoc* **53**: 457–481

Kim JP (1999) Surgical results with immunochemotherapy. In: Kim JP, Min JS, Mok YJ eds. Proceedings of the 3rd International Gastric Cancer Congress, Seoul 1999. Monduzzi Editore, Bologna (Italy), pp 3–9

Maehara Y, Hasuda S, Koga T, Tokunaga E, Kakeji Y and Sugimachi K (2000) Postoperative outcomes of sites of recurrence in patients following curative resection of gastric cancer. *Br J Surg* **87**: 353–357

Marujama K, Okabayashi K and Kinoshita T (1987) Progress in gastric cancer surgery in Japan and its limits of radicality. *World J Surg* **11**: 418–425

Nakajima T, Nashimoto A, Kitamura M, Kito T, Iwanaga T, Okabayashi K, Goto M and the Gastric Cancer Surgical Study Group (1999) Adjuvant mitomycin and fluorouracil followed by oral uracil plus tegafur in serosa-negative gastric cancer: a randomized trial. *Lancet* **354**: 273–277

Nishi M, Omori Y, Miwa K, eds (1995) Japanese classification of gastric carcinoma. Japanese Research Society for Gastric Cancer (JRSGC). 1st English edition. Tokyo: Kanehara & Co.

Roukos DH (2000a) Current status and future perspectives in gastric cancer management. *Cancer Treat Rev* **26**: 243–255. Review

Roukos DH (2000b) Extended (D2) lymph node dissection for gastric cancer: do patients benefit? *Ann Surg Oncol* **7**: 253–255. (editorial).

Roukos D, Hottenrott C, Lorenz M and Koutsogiorgas-Couchell S (1990) A critical evaluation of effectivity of extended lymphadenectomy in patients with carcinoma of the stomach. *J Cancer Res Clin Oncol* **116**: 307–313

Roukos DH, Lorenz M and Encke A (1998) Evidence of survival benefit of extended lymphadenectomy in Western gastric cancer patients based on a new concept. A prospective long-term follow-up study. *Surgery* **123**: 573–578

Roukos DH, Paraschou P and Lorenz M (2000) Distal gastric cancer and extensive surgery: a new evaluation method based on the study of the status of residual lymph nodes after limited surgery. *Ann Surg Oncol* **7**: 719–726

Sackett DL Rosenberg WMC, Gray JAM, Haynes RB and Richardson WS (1996) Evidence based medicine: what it is and what it isn't. *BMJ* **312**: 71–

Siewert JR, Boettcher K, Stein HJ and Roder JD (1998) Relevant prognostic factors in gastric cancer. Ten-year results of the German Gastric cancer Study. *Ann Surg* **228**: 449–461

The Lancet (Editorial) (2000) Overoptimisims about cancer. *Lancet* **355**: 157.

Wanebo HJ, Kennedy BJ, Chmiel J, Steele G, Winchester and Osteen R (1993) Cancer of the stomach: a patient care study by the American College of Surgeons. *Ann Surg* **218**: 583–587

Zippelius A, Pantel K (2000) RT-PCR-based detection of occult disseminated tumor cells in peripheral blood and bone marrow of patients with solid tumors. An overview. *Ann N Y Acad Sci* **20** (996): 110–123.

*© 2001 Cancer Research Campaign*
